# Supplementary figures and images for: Impact of seasonal malaria chemoprevention timing on clinical malaria incidence dynamics in the Kedougou region, Senegal
Source: PLOS Glob Public Health. 2025 Jan 15;5(1):e0003197. doi: 10.1371/journal.pgph.0003197 (PMC11734993; doi:10.1371/journal.pgph.0003197)

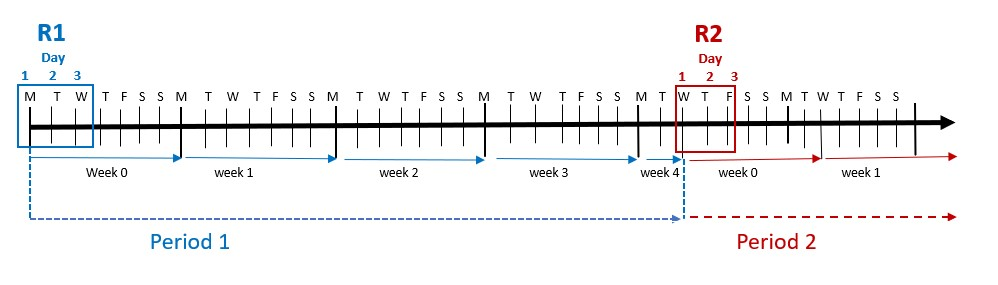

Supplement: S1 Fig — The SMC round number is represented by the letter R followed by the round number. Each round has 3 days of directly observed treatment. Week 0 is the week in which SMC was administered. The “SMC period” variable was defined as the interval between SMC rounds. For instance, SMC period 1 corresponds to the time between round 1 and round 2. Between two rounds, the maximum duration of a period was 36 days. The duration of SMC period 4, corresponding to the last round of SMC was therefore set up to week 5. (TIFF) [file pgph.0003197.s001.tiff]

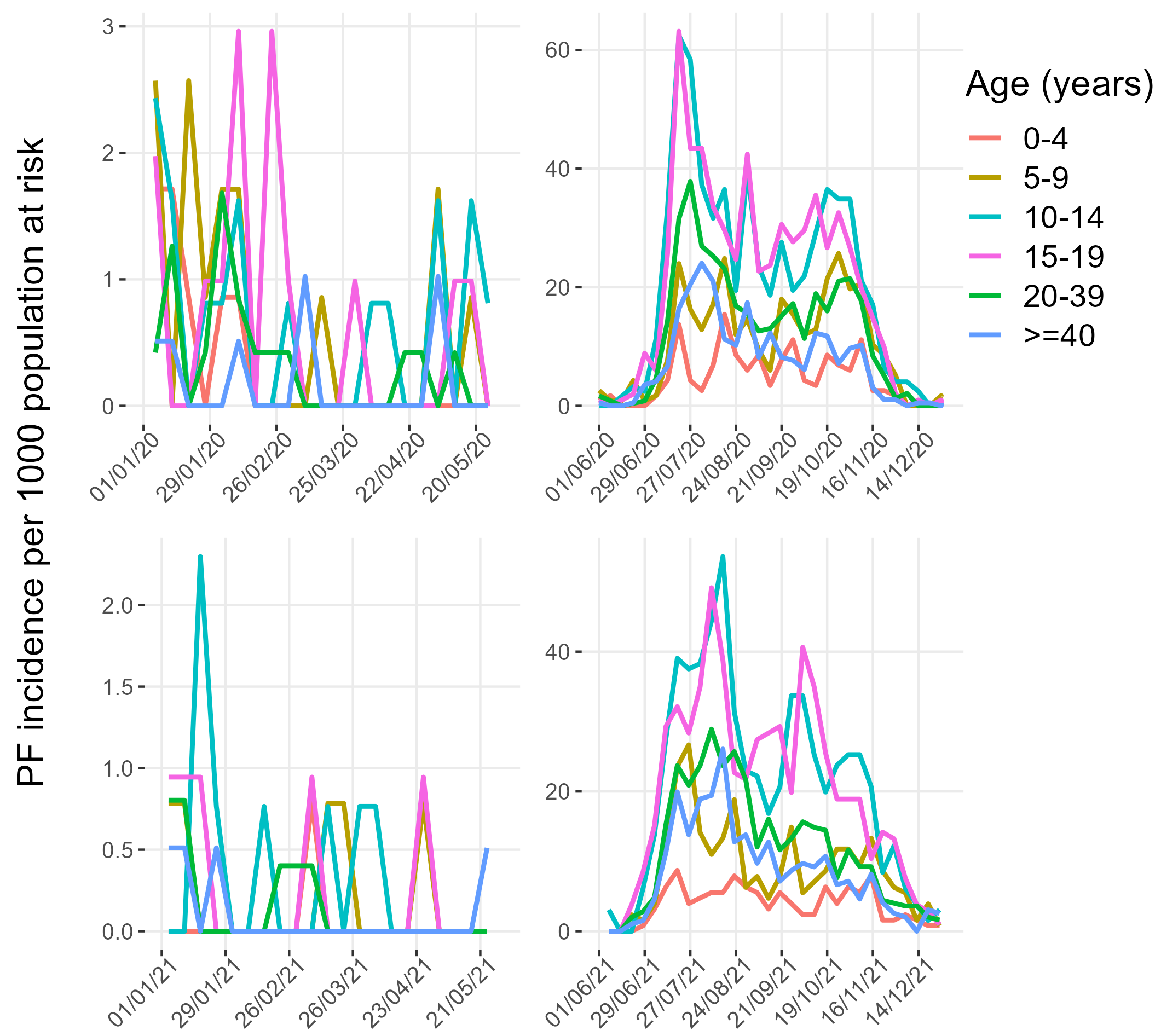

Supplement: S2 Fig — The scale of the y-axis changes differs across panels. (TIFF) [file pgph.0003197.s002.tiff]

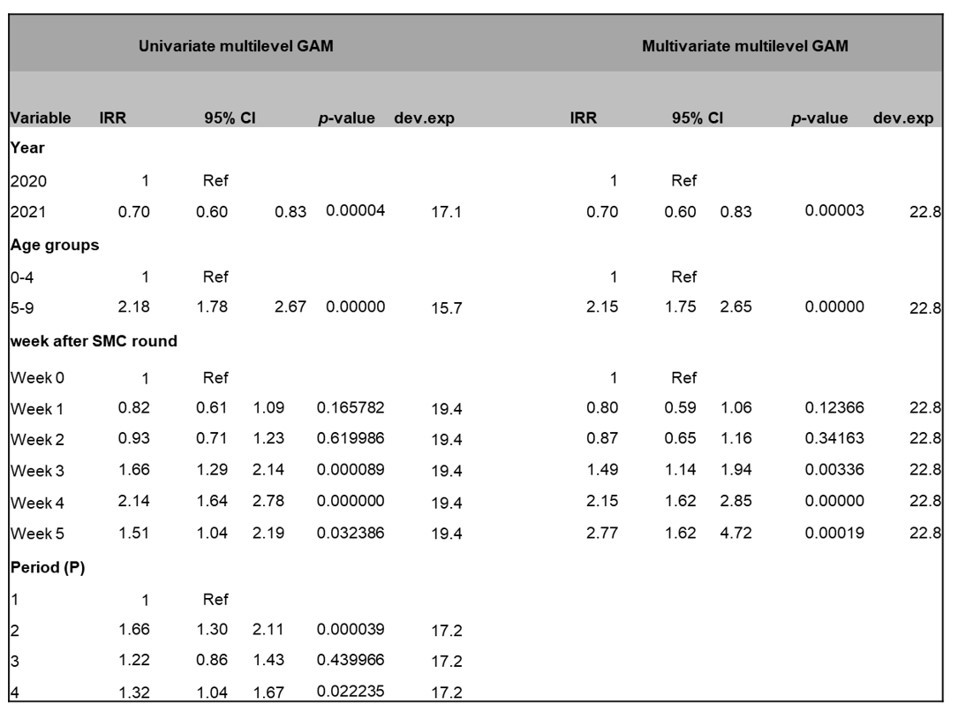

Supplement: S1 Table — (TIFF) [file pgph.0003197.s003.tiff]

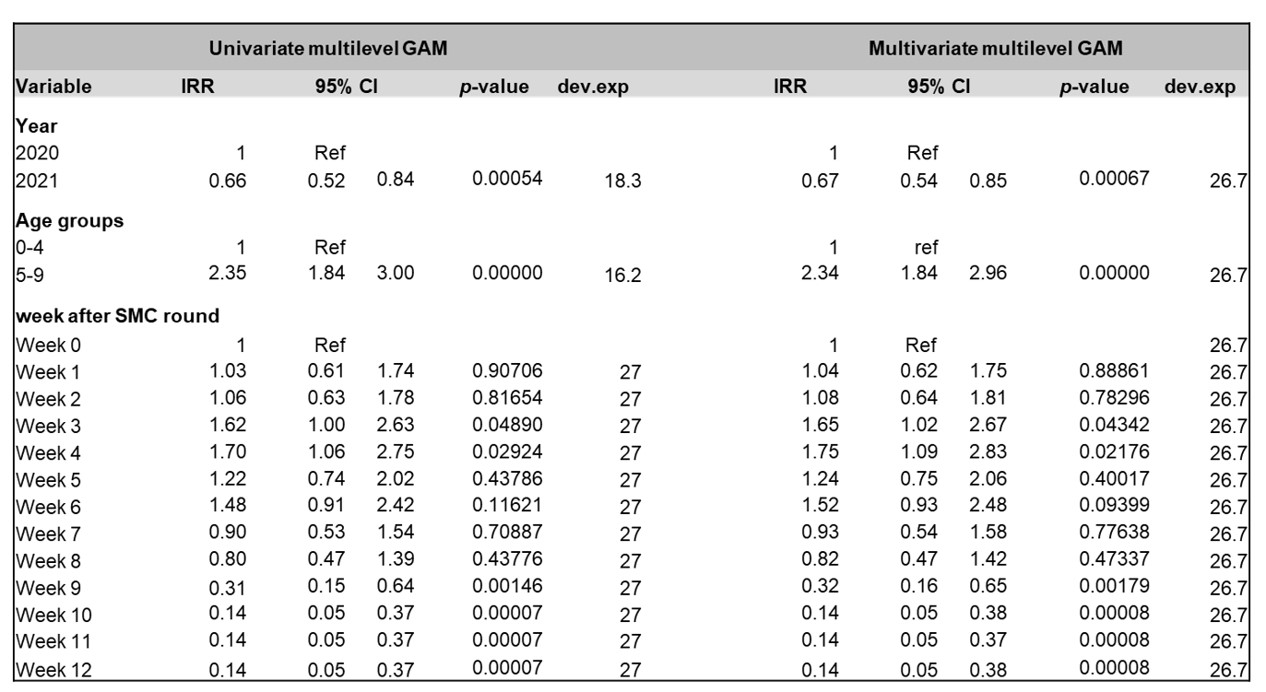

Supplement: S2 Table — (TIFF) [file pgph.0003197.s004.tiff]
